# Effects of a Taekwondo-Specific High-Intensity Interval Training Protocol on the Biomarkers of Cardiovascular and Biochemical Recovery in Collegiate Athletes

**DOI:** 10.7150/ijms.115788

**Published:** 2025-09-29

**Authors:** Po-Teng Huang, Chih-Han Wu, Po-Fang Huang, Ming-Chin Tsai, Kuei-Ling Chang, Kun-He Lin, Hsing-Chieh Huang, Shu-Cheng Lin

**Affiliations:** 1 Department of Physical Education and Sport Sciences, National Taiwan Normal University, Taipei, Taiwan.; 2 Office of Physical Education, Fu Jen Catholic University, New Taipei City, Taiwan.; 3 Office of Physical Education, National Central University, Taoyuan City, Taiwan.; 4 Department of Physical Education, Fu Jen Catholic University, New Taipei City, Taiwan.; 5 Department of Sports, Health and Leisure, Chung Hwa University of Medical Technology, Tainan City, Taiwan.; 6 School of Physical Education, Minnan Normal University, Zhangzhou, Fujian 363000, China.; 7 School of Gerontology and Long-Term Care, College of Nursing, Taipei Medical University, Taipei, Taiwan.

**Keywords:** physical training, lactic acid, creatine kinase, heart rate, competition

## Abstract

Taekwondo is a combat sport that requires a specific skillset and high physical fitness. In 2022, the rules of taekwondo competitions rules were revised to increase match intensity. In this study, we developed and validated a high-intensity interval training (HIIT) protocol for taekwondo athletes and compared the outcomes of this protocol with those of regular training (control). A total of 12 athletes were randomly assigned to either an HIIT group or a control group. Body mass index, heart rate (HR), lactic acid level, and creatine kinase level were measured both before and after relevant intervention. Our HIIT protocol consisted of two phases. The first phase (weeks 1 and 2) involved six rounds of four kicking techniques (roundhouse, back, axe, and spinning kicks), each performed for 21 s with 12-s rest (work [exercise]-to-rest ratio: 1.75:1). The second phase (weeks 3 and 4) involved eight rounds of the same kicking techniques, each performed for 24 s with 8-s rest (work-to-rest ratio: 3:1). The participants rested for 1 min between two rounds. The kicks were performed at 85% and 90% of the maximum HR. Significant (*p* ≤ 0.05) between-group differences were observed in HR at work, HR at rest, lactic acid level, and creatine kinase level. Overall, this study indicates that implementing a sport-specific HIIT protocol can enhance the recovery of HR and lactic acid and creatine kinase levels in taekwondo athletes.

## Introduction

Taekwondo is a combat sport that requires specific skills, advanced techniques, and high physical fitness. In 2022, the rules of taekwondo competitions were revised. The most notable change was the shift from a cumulative point system across three rounds to a format requiring competitors to win two out of three rounds. A round now ends when the point difference between competitors reaches 12 (as opposed to the previous threshold of 20 points). Furthermore, the current foul system allows a maximum of five fouls per round rather than across all three rounds. These modifications aimed to increase the intensity of competition, placing elevated physical demands on athletes. During each 2-min round, athletes attempt to win by landing punches and kicks on designated 1, 2. Bouts typically feature short high-intensity periods lasting 1-5 s, interspersed with longer nonfighting periods, with a phase ratio ranging from 1:2 to 1:7 1, 2. This work-to-rest ratio (WRR) resembles that of high-intensity interval training (HIIT), which involves repeated short (15-30 s) to long (2-4 min) bursts of high-intensity exercise, during which the heart rate (HR) reaches 80% to 100% of its maximum value (HRmax), followed by recovery periods lasting 6-240 s. In taekwondo competitions, the WRR generally ranges from 1:1 to 3:1 3.

Numerous studies have investigated the benefits of HIIT in various combat sports, such as karate 4, judo 5-8, taekwondo 9, Muay Thai 10, Brazilian jiu-Jitsu 11, and wrestling 12. Corresponding protocols varied in terms of intensity, ranging from 80% of the maximal aerobic capacity to all-out effort. The training programs were generally low in volume, lasted 4-8 weeks, and were progressively intensified between competitions 13, 14. Notably, HIIT stimuli in combat sports last longer than the typical effort-pause ratios observed in taekwondo matches 13, 15, 16. Many combat sports incorporate running as a mode of exercise. Multiple studies have explored sport-specific action patterns during HIIT 5, 6, 17, 18. For instance, a 4-week boxing HIIT program (3 rounds of 14 sets, consisting of 3 s of all-out punching with 10 s of rest between sets and 1 min of rest between rounds) increased peak power and oxygen consumption 18. A simulated boxing match enhanced punching force and the ability to maintain punching frequency. Similarly, a 4-week Judo HIIT protocol incorporating sport-specific techniques, such as the special judo fitness test, increased both upper and lower body peak power 5, 6. Despite these findings, few sport-specific training strategies have been developed to enhance performance in combat sports.

Cardiorespiratory endurance has long been recognized as a key component of physical fitness. Resting HR and lactic acid (LA) level are common biomarkers of fatigue, with shortened recovery times and increased HR and LA levels indicating elevated physical activity and exertion 19-21. Creatine kinase (CK) is an enzyme that leaks into the bloodstream when muscles and sarcolemmal structures are damaged 22. Physical exercise typically increases the levels of CK, which in turn reduces force production, maximal isometric force, and jump height 23, 24. In this study, we investigated the effects of a taekwondo-specific HIIT protocol on the biomarkers of cardiovascular and biochemical recovery in collegiate athletes. For this, we analyzed HR, LA level, and CK level as the indicators of HIIT outcome. We hypothesized that our HIIT protocol would outperform regular training in enhancing the recovery of HR, LA level, and CK level in taekwondo athletes.

## Methods

### Participants

A total of 12 taekwondo athletes voluntarily participated in this study. The study protocol was approved by the Human Research Ethics Committee of Fu Jen Catholic University, Taiwan (approval number: C104068). This study adhered to the ethical standards outlined in the Declaration of Helsinki. All participants were informed of the potential risks associated with this study, and written informed consent was obtained from each individual before enrollment. Table 1 presents detailed information on the participants and their training backgrounds.

### Experimental design

**Figure 1:**
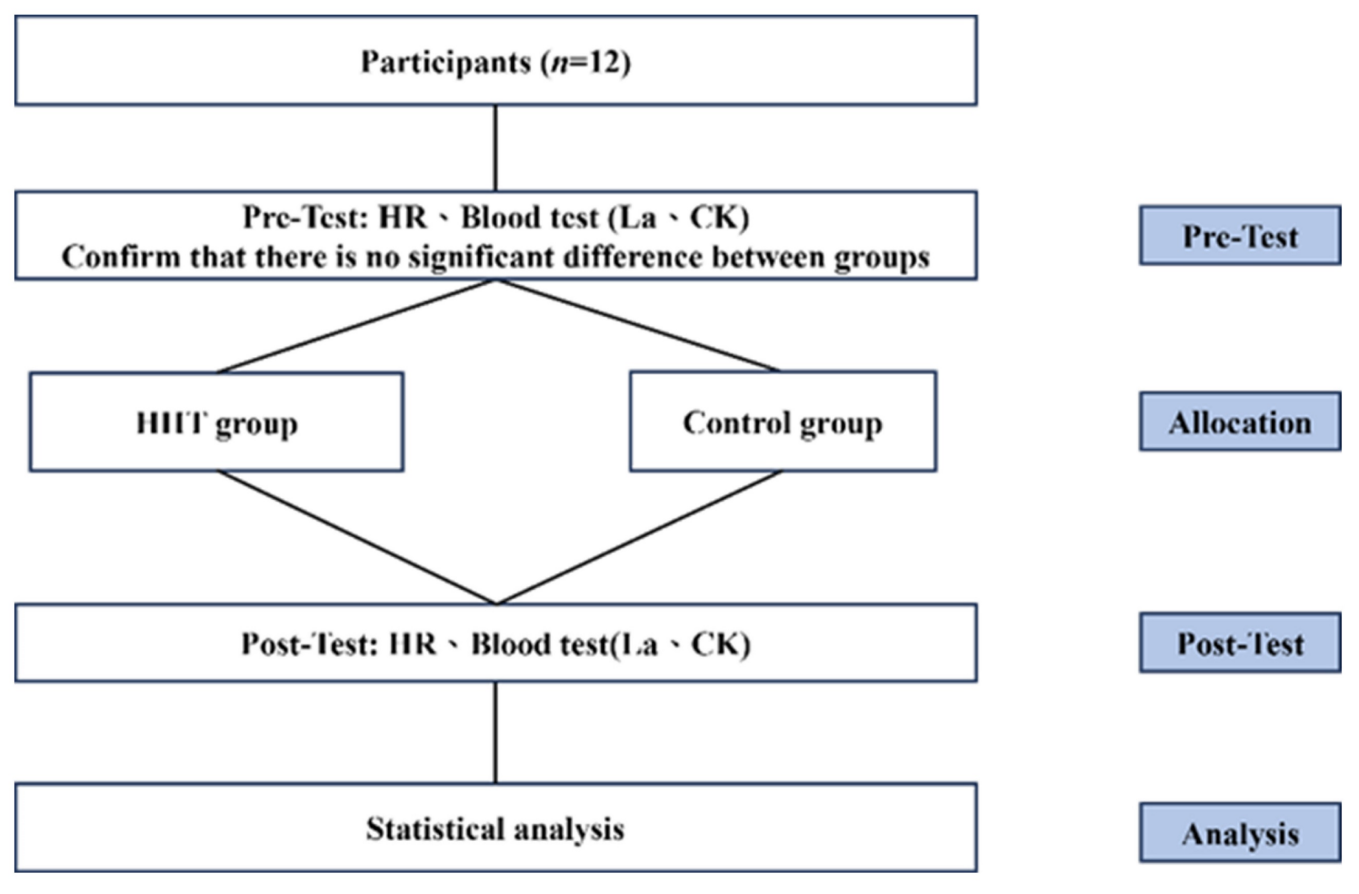
Experimental design.

The participants were randomly assigned to either a control group or an HIIT group. The control group received regular training, whereas the HIIT group received taekwondo-specific HIIT. Body mass index (BMI), HR, CK level, and LA level were measured both before and after the intervention. The participants were instructed to not engage in any additional physical training beyond their regular taekwondo training (Figure 1).

### Training interventions

Both groups completed 40 training sessions over a period of 4 weeks. The control group engaged in a regular taekwondo training session every morning and afternoon (approximately 90 min per session), five times a week. Each session included 10 min of warm-up, 60 min of technical drills and sparring practice, and 20 min of conditioning and cooldown. In these sessions, the training intensity was set to moderate, corresponding to approximately 70% to 80% of HRmax, which reflects the typical training intensity reported in the literature 25. The HIIT group followed a specific regimen. Participants in this group were monitored using Polar HR monitors to assess exercise intensity.

The HIIT program consisted of two phases. In the first phase (weeks 1 and 2), the participants engaged in six rounds involving four kicking techniques (roundhouse, back, axe, and spinning kicks). Each technique was performed for 21 s, followed by 12 s of rest (WRR: 1.75:1; Table 2). A resting period of 1 min was ensured between two rounds. In this phase, the participants performed their kicks at 85% of their HRmax. In the second phase (weeks 3 and 4), the participants engaged in eight rounds involving the same four kicking techniques. Each technique was performed for 24 s, followed by 8 s of rest (WRR: 3:1). A resting period of 1 min was ensured between two rounds. In this phase, the participants performed their kicks at 90% of their HRmax.

### Measurement of BMI and HR

Height and weight were measured to the nearest 0.1 cm and 0.1 kg, respectively. Measurements were performed with the participant in an upright position, barefoot, and in minimal clothing. Then, BMI was calculated using the measurements.

To measure resting HR, the participant was asked to lay down for 5 min, after which HR was measured using a Polar HR monitor. HRmax was estimated using the formula 220 - age, which is commonly applied in exercise physiology research 26.

### Assessment of blood biochemistry

A licensed nurse collected blood samples both before and after the training sessions. The samples were used to measure the levels of LA and CK. The blood level of LA was measured using a Biosen C-Line blood analysis system (EKF Diagnostics, Barleben, Germany). In brief, 10 μL of capillary blood was collected and mixed with a red blood cell lysis reagent. The mixture was then stored at a low temperature until analysis. Before analysis, instrument standardization and test calibration were performed; the coefficient of variation was ≤ 1.5%. The detection range for blood LA was set to 0.5-40 mmol/L. Analysis was performed using a fully automated dry chemistry analyzer (Fuji Dri-Chem 4000i; Fuji, Tokyo, Japan). To measure the blood level of CK, 10 µL of plasma was used. The detection range for CK was set to 10-2000 U/L.

### Statistical analysis

All statistical analyses were performed using SPSS (version 21; IBM, Armonk, NY, USA). A priori and post hoc power analyses were conducted using G*Power, with the significance level set at α = 0.05 and the effect size determined on the basis of a previous study 27. Statistical power (1 - β) reached 0.8, indicating that the sample size was reasonable. Data are presented as mean and standard deviation values. The study variables were analyzed using Wilcoxon's signed-rank test and the Mann-Whitney U test to compare pretest and posttest results within and between the two groups. Statistical significance was set p ≤ 0.05.

## Results

### HR

Table 3 presents the descriptive data on HR. In the HIIT group, significant differences were observed between pretest and posttest measures for R1HR, R2HR, and R3HR. However, in the control group, no significant pretest-posttest differences were noted. Significant differences were observed in posttest measures (PostR1HR, PostR2HR, and PostR3HR), but not pretest measures. Regarding exercise HR, the HIIT group exhibited significant pretest-posttest differences at all time points—E1, E3, E5, E7, and E9 (E1HR, E3HR, E5HR, E7HR, and E9HR). However, the control group exhibited significant pretest-posttest differences only at E5, E7, and E9, but not at E1 or E3. Posttest, but not pretest, comparisons revealed significant differences in exercise HR at all time points (PostE1HR, PostE3HR, PostE5HR, PostE7HR, and PostE9HR).

### Blood biochemistry

Table 4 presents a summary of the blood biochemical indicators. In the HIIT group, significant postintervention changes were observed at E5, but not at E24. Posttest comparisons revealed significant differences at PostE5, but not PostE24. Within-group comparisons revealed significant postintervention changes at E5 and E24 in the HIIT group, but not in the control group. Furthermore, significant differences were observed at PostE5 and PostE24; however, no significant differences were observed in the pretest results.

## Discussion

After the rules of taekwondo were modified in 2022, the physical demands on athletes increased considerably, emphasizing the importance of muscle strength, endurance, recovery capacity, and cardiovascular fitness. HIIT is an exercise approach that involves alternating short bouts of intense activity with brief recovery periods. This approach has gained attention as an effective strategy for inducing broad physiological adaptation across various sports, including combat sports. HIIT offers several advantages in taekwondo, in which repeated explosive effort is required throughout the match. In the present study, we noticed that implementing a sport-specific HIIT protocol combined with regular taekwondo training significantly enhanced the recovery of HR, LA level, and CK level. Although our relatively small sample size and short intervention period limited precluded causal inferences, the findings indicate meaningful associations between HIIT and improved recovery outcomes.

Before the rules of taekwondo were changed, athletes used to conserve their energy for the final round 28. However, after the implementation of the new regulations, athletes must sustainably exert their maximal effort in each round. Overall, the elevated HR, LA, and CK responses recorded during the HIIT sessions in our study confirm the high-intensity nature of the proposed protocol, and the reductions observed in these biomarkers and recovery time following training highlight its effectiveness. Notably, the HR, LA, and CK values achieved after training are similar to those reported in simulated matches 29, suggesting that the proposed protocol elicits physiological demands similar to those of competitions but with a minimal risk of injury. In taekwondo matches, the HR of competitors typically ranges between 178 and 193 bpm 28-31, with simulated matches having a similar range (172-197 bpm) 3, 15, 29, 32, 33. HR peaks in the third round 15, 28-34. The levels of LA are also similar between matches (6.1-14 mmol/L) and simulations (4.2-13.2 mmol/L) 28-37. An elevated level of CK, widely recognized as a biomarker of muscle damage and fatigue 38-42, has been associated with the impairment of neuromuscular function 6, 43, 44. For comparability, our control group engaged in structured taekwondo training of moderate intensity and standard duration, consistent with previously reported protocols 25.

Compared with the control group, the HIIT group exhibited rapid HR and LA level recovery, a practically relevant advantage in tournaments where athletes may engage in several bouts of competition every day. This finding is consistent with those of studies suggesting that HIIT enhances the transport and clearance of LA and can be incorporated into taekwondo-specific movements to reproduce sport-specific metabolic demands 45-47. Although the 21-24-s kicking segments in our protocol do not replicate every tactical element of competition, they align with the time-motion structure of taekwondo and are expected to stimulate aerobic-anaerobic adaptation 1, 32. Furthermore, our results indicated faster CK recovery in the HIIT group than in the control group; however, CK remains an indirect and highly variable marker influenced by training status, fiber type, genetics, and exercise modality. Hence, repeated bouts may further attenuate CK elevation with training. Therefore, our findings should be interpreted with caution. Future studies should triangulate CK with other biomarkers (e.g., myoglobin and lactate dehydrogenase), neuromuscular performance, and perceptual measures to facilitate a comprehensive evaluation 48-50. Improvements in cardiovascular fitness should be inferred primarily from submaximal HR responses and recovery, particularly because HRmax typically remains stable or slightly decreases with endurance training 51.

In summary, our protocol is designed to simulate the intermittent, high-intensity nature of taekwondo competitions. Although this protocol does not fully replicate tactical variability, the improvements observed in HR, LA, and CK recovery strongly suggest its potential benefits in competition readiness, particularly in the context of multibout tournaments. This suggestion is supported by randomized studies demonstrating that tailoring interval formats and WRRs with sport-specific techniques reduces peak HR and LA responses, improves aerobic fitness and agility, and enhances sports-specific performance 52-55. Although our sample size was limited to 12 participants, power analysis confirmed that it was sufficient to detect medium or large effects, indicating that our findings remain valuable and informative.

## Conclusion

In this study, we developed a sport-specific HIIT protocol to enhance the competitive performance of taekwondo athletes. Our results indicated that this protocol significantly improved the recovery of physiological indicators such as HR and LA and CK levels. Compared with conventional training, the proposed protocol was associated with rapid HR normalization, LA clearance, and muscle recovery. Taken together, these findings indicate that sport-specific HIIT is an effective and time-efficient approach for optimizing the recovery and performance of athletes, particularly during highly demanding competitions. To maximize its benefits, HIIT should be carefully tailored and periodized to align with individual athlete profiles and training cycles.

### Recommendations for Coaches and Athletes

Taekwondo coaches and athletes should implement sport-specific HIIT protocols because these protocols can effectively enhance LA clearance and HR and CK recovery. The training protocols can also improve cardiovascular endurance, reduce fatigue, and facilitate muscle repair, particularly before competitions. To maximize benefits and avoid overtraining, HIIT programs should be individualized and periodized depending on each athlete's specific needs, including the gradual progression of intensity and duration, coupled with appropriate low-intensity recovery sessions. Overall, our HIIT protocol significantly enhances HR, LA, and CK recovery over a short period and can thus serve as an effective strategy for accelerating physiological recovery in taekwondo athletes.

## Figures and Tables

**Table 1:** Participants' physical characteristics before the intervention.

Characteristics	High-intensity interval training group	Control group	*p* value
Age (years)	20.83 ± 0.60	20.67 ± 0.59	0.717
Height (cm)	166.67 ± 2.68	170.67 ± 2.11	0.188
Weight (kg)	62.47 ± 2.75	63.12 ± 2.02	0.816
Body mass index (kg/m^2^)	22.48 ± 0.57	21.72 ± 0.75	0.320
Training age (years)	13.83 ± 0.74	10.50 ± 1.31	0.355
Heart rate (bpm)	57.83 ± 2.48	55.17 ± 1.72	0.052
Lactic acid (mmol/L)	1.01 ± 0.16	0.98 ± 0.12	0.743
Creatine kinase (U/L)	113.17 ± 11.00	102.83 ± 6.43	0.092

**Table 2:** HIIT training protocol.

		Week 1	Week 2	Week 3	Week 4
HIIT	Morning	6 rounds 1 kick 20 s/10 s	6 rounds 1 kick 20 s/10 s	8 rounds 1 kick 24 s/8 s	8 rounds 1 kick 24 s/8 s
	Afternoon	6 rounds 1 kick 20 s/10 s	6 rounds 1 kick 20 s/10 s	8 rounds 1 kick 24 s/8 s	8 rounds 1 kick 24 s/8 s

1 kick 20 s/10 s: one type of kick for 20 s, followed by 10 s of rest. 1 kick 24 s/8 s: one type of kick for 24 s, followed by 8 s of rest. The participants rested for 1 min between two rounds. Abbreviations**:** HIIT, high-intensity interval training.

**Table 3:** Descriptive data on HR.

Performance	Test	HIIT group		Control group	
		Before training	After training	Before training	After training
HR at rest HR at work (exercise)	HR0	57.83 ± 2.48	57.33 ± 1.75	55.17 ± 1.72	55.83 ± 0.75
	R1HR	172.17 ± 2.32^a^	157.50 ± 4.51^a,b^	172.17 ± 2.14	170.17 ± 1.72^b^
	R2HR	180.17 ± 3.19^a^	169.67 ± 3.50^a,b^	178.83 ± 3.87	176.67 ± 5.05^b^
	R3HR	187.50 ± 2.88^a^	172.67 ± 3.33^a,b^	184.17 ± 2.86	182.17 ± 3.06^b^
HR at rest	E1HR	153.50 ± 3.02^a^	126.83 ± 2.93^a,b^	154.83 ± 3.87	154.33 ± 6.28^b^
	E3HR	114.17 ± 3.97^a^	84.67 ± 3.61^a,b^	113.17 ± 2.48	109.83 ± 3.66^b^
	E5HR	105.17 ± 4.02^a^	80.67 ± 2.58^a,b^	109.83 ± 1.94^a^	96.33 ± 2.06^b,c^
	E7HR	96.33 ± 4.32^a^	74.50 ± 2.17^a,b^	104.67 ± 3.27^a^	93.17 ± 3.13^b,c^
	E9HR	91.17 ± 3.97^a^	72.33 ± 1.75^a,b^	96.50 ± 3.62^a^	89.33 ± 1.97^b,c^

^a^Difference between the pretest and posttest results in the HIIT group; ^b^Postintervention changes; ^c^Difference between the pretest and posttest results in the control group. R1, R2, and R3 refer to rounds 1, 2, and 3, respectively. E1, E3, E5, E7, and E9 refer to 1, 3, 5, 7, and 9 min after rest, respectively. Abbreviations: HIIT, high-intensity interval training; HR, heart rate.

**Table 4:** Descriptive data on blood biochemistry.

Performance	Test	HIIT group		Control group	
		Before training	After training	Before training	After training
Lactic acid	E5	6.43 ± 0.33^a^	3.57 ± 0.26^a,b^	7.13 ± 0.26	7.55 ± 0.27^b^
	E24	1.20 ± 0.14	1.28 ± 0.12	1.41 ± 0.15	1.38 ± 0.15
Creatine kinase	E5	364.17 ± 38.88^a^	234.67 ± 37.23^a,b^	360.83 ± 28.35	349.33 ± 24.41^b^
	E24	211.67 ± 26.73	138.67 ± 10.31	197.17 ± 16.25	198.67 ± 14.07

^a^Difference between the pretest and posttest results in the HIIT group; ^b^Postintervention changes; ^c^Difference between the pretest and posttest results in the control group. E5: After 5 min of rest. E24: After 24 h of rest. Abbreviation**:** HIIT, high-intensity interval training.
